# Occupational Distress Among Nurses Through Social Media Narratives: A Scoping Review

**DOI:** 10.3390/healthcare14193146

**Published:** 2026-09-22

**Authors:** Giovanni Gioiello, Dhurata Ivziku, Mariana Alina Renghea, Angélica Isabel Romero Daza, Marika Lo Monaco, Giuliano Anastasi, Massimo Tusconi, Felice Curcio, Marzia Lommi, Cesar Ivan Aviles Gonzalez

**Affiliations:** 1 Department of Medicine and Surgery, University of Enna “Kore”, 94100 Enna, Italy; giovanni.gioiello@unikore.it (G.G.); giuliano.anastasi@unikore.it (G.A.); cesar.aviles@unikore.it (C.I.A.G.); 2 Department of Health Professions, Fondazione Policlinico Universitario Campus Bio-Medico, 00128 Rome, Italy; d.ivziku@policlinicocampus.it; 3 Faculty of Medical Science, AAB College, 10000 Pristina, Kosovo; 4 Faculty of Health Sciences and Faculty of Medicine, Francisco de Vitoria University, 28223 Madrid, Spain; 5 Faculty of health, Popular University of Cesar, Valledupar 200002, Colombia; angelicaromero@unicesar.edu.co; 6 Department of Maternal and Child Health Promotion, Internal Medicine & Specialties of Excellence “G. D’Alessandro” (PROMISE), University of Palermo, 90127 Palermo, Italy; marika.lomonaco@unipa.it; 7 Center of Liason Psychiatry and Psychosomatics, University Hospital of Cagliari, 09042 Cagliari, Italy; massimotusconi@yahoo.com; 8 Faculty of Medicine and Surgery, University of Sassari, 07100 Sassari, Italy; 9 Department of Biomedicine and Prevention, University of Rome Tor Vergata, 00133 Rome, Italy; marzia.lommi@uniroma2.eu

**Keywords:** nurses, occupational stress, burnout, psychological distress, social media, workload, professional identity, organizational factors, review

## Abstract

**Highlights:**

**What are the main findings?**

- Nurses’ social media narratives included expressions of occupational distress and adjacent work-related constructs, including stress, emotional exhaustion, anxiety, frustration, anger, and isolation, while burnout emerged as a related or potential occupational outcome.
- Organizational and work-environment factors, including staffing shortages, excessive workload, insufficient resources, workplace violence, limited autonomy, and inadequate support, recurred within narratives of work-related difficulties, without implying statistical associations or causality.

**What are the implications of the main findings?**

- Nurse-generated social media content may complement conventional research methods by capturing spontaneously expressed work-related concerns and professional experiences, while remaining subject to self-selection and representativeness limitations.
- The findings provide a descriptive mapping of organizational conditions and professional experiences represented in nurses’ social media narratives, without supporting inferences about prevalence, causality, or intervention effectiveness.

**Abstract:**

**Background/Objectives:** Occupational distress threatens nurses’ well-being, workforce sustainability, and quality of care. Although most evidence derives from questionnaires, interviews, and other researcher-defined methods, nurses increasingly use social media to communicate work-related experiences. This review mapped the evidence on occupational distress represented in nurse-generated social media content, its main dimensions and organizational sources, and the role of social media in professional expression and self-representation. **Methods:** A scoping review was conducted according to Joanna Briggs Institute methodology and reported following PRISMA-ScR guidance. PubMed, CINAHL Complete, and Scopus were searched. Primary qualitative, quantitative, and mixed-methods studies analyzing social media content attributed to nurses according to the procedures reported in the primary studies were eligible. Findings were synthesized descriptively and narratively. **Results:** Nine articles published between 2018 and 2023 were included. They examined blogs, Facebook, professional nursing websites, Sina Microblog/Weibo, Twitter, Instagram, AllNurses, and Reddit using qualitative, mixed-methods, and computational approaches. Occupational distress and relevant work-related experiences were represented through psychological and emotional, physical, social/family, and professional-organizational manifestations. Nurses’ social media narratives included expressions of occupational distress and adjacent work-related constructs, such as stress, burnout, emotional exhaustion, anxiety, frustration, anger, and isolation. Recurrent organizational factors included staffing shortages, excessive workload, insufficient resources, workplace violence, limited professional autonomy, inadequate recognition, compensation and rights, professional agency, safety, and organizational support. Most evidence was generated during the COVID-19 pandemic. Social media was also represented as spaces for narration of professional experiences, support, public communication and advocacy, and professional self-representation. **Conclusions:** Nurse-generated social media content provides a complementary perspective on how occupational distress is experienced, interpreted, and communicated outside researcher-defined settings. The available evidence remains limited, heterogeneous, and strongly influenced by the COVID-19 pandemic. Social media content represents situated digital narratives rather than a representative proxy for the nursing workforce and does not support inferences about prevalence or causality. More geographically diverse, post-pandemic, and methodologically integrated research using independent datasets is needed.

## 1. Introduction

Nursing workforce sustainability is a major global health system priority [1]. Nurses constitute a critical component of healthcare delivery, yet persistent workforce shortages, retention difficulties, and inequalities in workforce distribution continue to challenge health systems internationally [2]. Nurses’ occupational well-being is therefore relevant to quality of care, patient safety, service continuity, and workforce retention [3,4].

Occupational distress has been conceptualized in nursing as a multidimensional, work-related phenomenon characterized by sustained psychological, emotional, and professional strain arising from adverse or inadequately resourced working conditions [5,6,7]. Although closely related to work-related stress, burnout, moral distress, moral injury, compassion fatigue, and psychological distress, occupational distress is not equivalent to these constructs [5,8,9]. Recent conceptual work describes persistent emotional strain, a sense of powerlessness, psychological and physical manifestations, and systemic and organizational influences as key features of occupational distress [5]. This perspective also helps distinguish occupational distress from its possible antecedents and consequences. Conditions such as understaffing, high workload, limited autonomy, and insufficient organizational support may contribute to occupational distress, whereas burnout, professional disengagement, and intention to leave may emerge as related or subsequent outcomes. Importantly, occupational distress should not be understood solely in terms of individual vulnerability, as it is closely embedded in the structural and organizational conditions in which nurses work [5].

The Job Demands–Resources (JD-R) model provides a useful theoretical framework for understanding these processes [10,11]. According to the model, sustained exposure to high job demands in the presence of insufficient personal, professional, or organizational resources can progressively impair occupational well-being. In nursing, workload, time pressure, understaffing, exposure to suffering, role conflict, and workplace violence are recognized job demands associated with adverse occupational experiences and outcomes [12,13,14,15]. Conversely, supervisor and peer support, effective leadership, professional autonomy, recognition, and adequate resources may help mitigate the impact of these demands [16,17,18].

The JD-R model therefore offers a useful framework for considering the interplay between working conditions, available resources, expressions of distress, and potential occupational outcomes while keeping these dimensions conceptually distinct.

Most evidence on nurses’ occupational distress has been generated through questionnaires, interviews, focus groups, and other researcher-defined methods [8,19,20].

Although these approaches are essential for measuring occupational outcomes and exploring nurses’ experiences, they capture data within predefined research contexts. In parallel, social media have become relevant spaces in which nurses communicate about clinical practice, working conditions, professional concerns, and emotional experiences [21,22].

Unlike data collected within a specific research encounter, much social media content is generated spontaneously as part of everyday digital communication. It may therefore complement traditional qualitative approaches by providing access to narratives, self-representations, and discursive practices that emerge outside formal data-collection procedures [23,24]. During the COVID-19 pandemic, research examining nurses’ social media content expanded rapidly, using approaches such as qualitative content analysis, thematic analysis, sentiment analysis, topic modelling, and natural language processing [25,26,27].

Despite this growing body of research, the evidence remains fragmented. Previous reviews have examined nurses’ use of social media during the COVID-19 pandemic [21], methodological approaches to social media analysis in healthcare research [25], and the application of natural language processing to social media data in nursing research [27]. However, these perspectives do not provide an integrated account of how occupational distress is represented in social media content generated by nurses across different platforms.

The literature is also characterized by conceptual and terminological heterogeneity, as studies may address related work experiences without explicitly using the term “occupational distress.”

As a result, there is limited understanding of how occupational distress is expressed in nurse-generated social media narratives, which dimensions of distress emerge from these narratives, and which organizational factors and working conditions are described alongside them. It also remains unclear how nurses use different social media platforms to express work-related experiences, interact with and support others, and represent themselves professionally.

Therefore, the review was guided by the following research questions:

1. How is occupational distress represented in nurses’ social media narratives, and which dimensions emerge from these narratives?
2. Which organizational factors and working conditions are described by nurses as potential sources of occupational distress or co-occur within narratives of distress?
3. How are social media used by nurses as spaces for expressing work-related experiences, interaction and support, as well as for professional self-representation?

## 2. Materials and Methods

### 2.1. Study Design

A scoping review was conducted following the methodological framework proposed by Arksey and O’Malley [28] and the Joanna Briggs Institute (JBI) guidance for conducting scoping reviews [29,30]. The review protocol was registered on the Open Science Framework (OSF; osf.io/td4b9). Following protocol registration, methodological refinements were made to the search approach, including changes to the information sources, language eligibility, and the addition of supplementary citation searching.

Reporting was conducted in accordance with the *Preferred Reporting Items for Systematic Reviews and Meta-Analyses Extension for Scoping Reviews* (PRISMA-ScR) [31]. The completed PRISMA-ScR checklist is provided in the Supplementary Materials.

### 2.2. Eligibility Criteria

Eligibility criteria were defined according to the Population–Concept–Context (PCC) framework and refined through an initial exploratory search, consistent with scoping review methodology [28,29,30,31]. The preliminary search was used to familiarize the research team with the terminology, source types, and range of experiences represented in the literature. The inclusion and exclusion criteria are summarized in Table 1.

Eligible sources included primary quantitative, qualitative, and mixed-methods studies published as articles in scientific journals. For the purposes of this review, “nurse-generated social media content” was defined as textual, audiovisual, or multimodal content produced directly by users identified in the original studies as nurses or members of the nursing profession. Eligibility was not restricted by nursing specialty, healthcare setting, or level of professional experience. Because professional titles and nursing roles vary across countries and studies, eligibility was based on the professional classification reported by the primary-study authors rather than on a predefined list of nursing job titles. Nursing students were excluded. In multiprofessional studies, only data specifically attributable to nurses and amenable to separate analysis were eligible.

Given the conceptual and terminological heterogeneity of the literature, the Concept was operationalized broadly while maintaining a distinction between phenomena located at different conceptual levels. Accordingly, three categories were distinguished: (a) expressions of distress and adjacent work-related constructs, such as occupational stress/work-related stress, emotional exhaustion, psychological distress, moral distress, moral injury, compassion fatigue, and frustration; (b) working and organizational conditions described as sources or contributing factors, including workplace violence or discrimination, staffing shortages, workload, and limited professional autonomy or recognition; and (c) related constructs or potential occupational outcomes, such as burnout, job dissatisfaction, and intention to leave the job or profession. These phenomena were not considered interchangeable components of occupational distress.

The explicit presence of the term occupational distress was not required for inclusion. Studies using adjacent terminology were considered potentially eligible when they examined nurses’ work-related experiences consistent with the Concept of the review and met the other eligibility criteria. The inclusion of these constructs was therefore intended to enhance conceptual sensitivity and did not imply that they were considered synonymous with occupational distress. Studies examining social media as spaces for professional narration, peer support, or professional self-representation were also considered eligible when these dimensions were analyzed in relation to occupational distress or relevant work-related experiences.

No language filters were applied during the database searches. At the study-selection stage, eligibility was restricted to articles published in English or Italian, reflecting the languages that could be directly assessed by the review team. Grey literature was not included. Reviews, editorials, commentaries, and letters to the editor were also excluded.

### 2.3. Information Sources and Search Strategy

The initial literature search was conducted from 15 May 2026 in PubMed, CINAHL Complete, and Scopus. The search strategies were subsequently updated and rerun on 2 September 2026; the results of the most recent search were used for the final study selection process and are reported in Supplementary Table S1.

PubMed was selected for its broad coverage of biomedical and health-sciences literature, CINAHL Complete for its specific coverage of nursing and allied-health literature, and Scopus to provide broader multidisciplinary coverage relevant to occupational health and digital/social media research.

The search strategy combined controlled vocabulary, where available and appropriate for the specific database, free-text terms, and Boolean operators. The strategies were adapted to the indexing system, syntax, and search fields of each database.

The strategy was structured around three main conceptual blocks: nursing professionals; social media and digital platforms; and occupational distress and relevant work-related phenomena.

The occupational distress block was intentionally formulated broadly to maximize search sensitivity and identify studies describing work-related distress using terminology other than “occupational distress.” Terms referring to adjacent constructs, organizational conditions, and potential outcomes were used for information retrieval purposes and not as synonyms or conceptual equivalents of occupational distress.

The complete search strategies actually executed for each database, including controlled vocabulary terms, free-text terms, field tags where used, database-specific syntax, phrase searching and truncation procedures, any limits applied, date of execution, and number of records retrieved, are reported in Supplementary Table S1.

The search strategy was not subjected to a formal peer-review or validation process using a standardized instrument.

In addition to the electronic database search, backward and forward citation searching was conducted for the articles included in the review. Backward citation searching was performed by examining the reference lists of the included articles, whereas forward citation searching was conducted using Google Scholar to identify subsequent publications citing the included articles. Records identified through citation searching were assessed using the same predefined eligibility criteria applied to the database searches; therefore, grey literature was not eligible for inclusion. The identification and selection process for records retrieved through citation searching is reported separately in the PRISMA flow diagram (Figure 1).

### 2.4. Selection of Sources of Evidence

Study selection was conducted in two stages. First, two reviewers (G.G. and C.I.A.G.) independently screened the titles and abstracts of all identified records against the predefined eligibility criteria. Second, the same reviewers independently assessed the full texts of potentially eligible studies.

Eligibility was not determined solely on the basis of the presence of the term “occupational distress.” For studies using adjacent constructs, relevance was assessed by jointly considering the work-related nature of the experience examined, whether the content pertained to the nursing population, its nurse-generated origin, and its consistency with the conceptual categories defined in the eligibility criteria.

In borderline cases, the two reviewers assessed the study against the full set of these criteria rather than solely on the terminology used by the authors. Disagreements were resolved through discussion and consensus; when consensus could not be reached, a third reviewer (F.C.) was consulted.

### 2.5. Data Charting Process

A standardized data-charting form was developed in Microsoft Excel for Microsoft 365 (Microsoft Corporation, Redmond, WA, USA) following JBI guidance [29,30]. Two authors (G.G. and C.I.A.G.) independently extracted data from each included source. Discrepancies were resolved through discussion. When agreement could not be reached, a third author (F.C.) was consulted.

Before final data extraction, the data-charting form was preliminarily tested on the first included studies to assess its completeness and applicability to the information reported in the eligible sources. This preliminary use informed minor refinements to the form, including the addition or clarification of fields required to capture the prespecified data items. The refined form was subsequently used for data extraction across all included articles.

### 2.6. Data Items

The following information was charted for each included study: reference; country; study design; study aim; social media platform; sample or dataset characteristics; and key findings.

When reported in the original studies, the following information was also collected: data-collection period, type of social media content analyzed, analytical approach, COVID-19 or non-COVID-19 context, methods used by the authors to identify content as nurse-generated, information on ethical procedures or data governance, and the main domains of occupational distress or work-related distress identified.

### 2.7. Critical Appraisal of Individual Sources of Evidence

Consistent with the exploratory purpose of a scoping review, no formal methodological quality or risk-of-bias appraisal was performed [28,29,30]. The objective was to map the nature, characteristics, and range of available evidence rather than to determine intervention effectiveness or establish causal relationships [31]. Accordingly, studies were not excluded based on methodological quality.

The absence of a formal methodological quality assessment was taken into account when interpreting the findings. Accordingly, the synthesis was not used to draw conclusions about the certainty of the evidence, estimate the prevalence of occupational distress, establish causal relationships, or evaluate the effectiveness of interventions.

### 2.8. Synthesis of Results

Consistent with the aims of this scoping review and the framework proposed by Arksey and O’Malley [28], findings were synthesized using a descriptive narrative approach [32,33]. Results were presented narratively and complemented by summary tables.

The synthesis proceeded in two stages. First, the descriptive and methodological characteristics of the included studies were summarized, including publication year, geographical context, social media platform, study design, dataset characteristics, and analytical approach.

Second, findings relevant to the review questions were compared across studies and organized into domains.

The Job Demands–Resources Model was used as an interpretive framework to organize, where applicable, the working conditions represented in the studies into job demands and job resources and to maintain their conceptual distinction from expressions of distress and potential occupational outcomes. The framework was applied for descriptive and interpretive purposes and was not used to infer statistical associations or causal relationships not demonstrated by the included studies. When multiple publications used partially overlapping datasets, the reports were retained separately to preserve the specific information provided by each publication; however, the overlapping component was not considered independent evidence in the synthesis.

## 3. Results

### 3.1. Study Selection Results

The electronic database searches identified 2342 records: 632 from PubMed, 829 from CINAHL Complete, and 881 from Scopus. After removal of 721 duplicate records, 1621 records underwent title and abstract screening, of which 1607 were excluded. Fourteen reports were sought for retrieval and all were successfully retrieved and assessed for eligibility. Eight reports were excluded at the full-text stage: seven because nurse-generated social media content was not separately analyzable and one because the professional identity of the social media content could not be attributed to nurses. Six articles identified through database searching met the eligibility criteria and were included.

Backward and forward citation searching identified an additional 658 records. Twelve reports were sought for retrieval and all were retrieved and assessed for eligibility. Nine reports were excluded at the full-text stage: seven because nurse-generated social media content was not separately analyzable and two because the professional identity of the social media content could not be attributed to nurses. Three additional articles identified through citation searching met the eligibility criteria.

Overall, nine articles were included in the final review. The study-selection process, including the separate identification pathways through database searching and citation searching, is presented in the PRISMA flow diagram (Figure 1).

### 3.2. Characteristics of the Included Publications

Nine articles published between 2018 and 2023 met the inclusion criteria. Six were identified through electronic database searching [26,34,35,36,37,38], whereas three additional articles were identified through backward and forward citation searching [39,40,41].

Although the final search was rerun in September 2026 without publication-date restrictions, no articles published after 2023 met the eligibility criteria.

The included publications examined nurse-generated content across blogs, Facebook and professional nursing websites, Sina Microblog/Weibo, Twitter, Instagram, AllNurses, and Reddit. They were heterogeneous in geographical context, data-collection period, dataset size and format, procedures used to identify nurse-generated content, and analytical approach. Most of the evidence was generated in the context of the COVID-19 pandemic. The main characteristics of the included publications and datasets are summarized in Table 2.

Fontanini et al. [37] and Rossettini et al. [41] were based on partially overlapping datasets collected during different waves of the COVID-19 pandemic. Fontanini et al. [37] analyzed 380 narratives collected during the first COVID-19 wave in Italy (23 February–3 May 2020). Rossettini et al. [41] incorporated the same 380 first-wave narratives and added 161 new narratives collected during the second/third COVID-19 waves (13 October 2020–23 May 2021), enabling longitudinal comparison across pandemic waves. Accordingly, findings derived from the shared first-wave corpus were not treated as independent evidence in the synthesis.

### 3.3. Representations and Dimensions of Occupational Distress

Occupational distress and relevant work-related experiences were represented through psychological and emotional, physical, social/family, and professional-organizational manifestations. These dimensions were not uniformly present across the nine publications and, given the partial overlap between Fontanini et al. [37] and Rossettini et al. [41], their presence should not be interpreted as a frequency of independent studies.

#### 3.3.1. Psychological and Emotional Manifestations

Castro and Andrews [34] identified *feeling stressed and burnt out* as one of the main themes among the four nurse bloggers, with a markedly different distribution of coded references across blogs; these frequencies described the textual material and not the prevalence of stress or burnout in the nursing population. Forte and Pires [39] reported content characterized by fear, fatigue, pressure, concern, distress, and insecurity during the early phase of the pandemic. Fontanini et al. [37] described sadness, fear, helplessness, insecurity, anxiety, anger, and exhaustion, while Chen et al. [35] identified *physical and psychological distress* alongside commitment, pride, growth, and self-support.

In the longitudinal follow-up by Rossettini et al. [41], *emotional ups and downs* continued to be represented during the second/third wave; the overall theme *Sharing what is happening within myself* decreased compared with the first wave, whereas no significant differences emerged for the subtheme concerning emotional fluctuations. Çiçek Doğan et al. [36] reported stress, fear of infection, worthlessness, depression, hopelessness, and mental exhaustion. Jiang et al. [26] identified *nurse stress and burnout* among the work concerns. Luo et al. [40] found, in addition to predominantly positive and neutral sentiments in the automated classification of posts, qualitative experiences including fear, burnout, disappointment, grievance, anxiety, concern, and helplessness. Finally, Milicia et al. [38] described the *Emotional Burden of Nursing*, including the emotional cost of care and situations of moral distress in Reddit comments.

#### 3.3.2. Physical Manifestations

Physical manifestations were particularly visible in pandemic-related studies. Forte and Pires [39] reported fatigue, pressure, and discomfort associated with the use of protective equipment. Fontanini et al. [37] described physical discomfort in relation to prolonged shifts, staff shortages, and PPE; Rossettini et al. [41] confirmed the persistence of the subtheme *dealing with physical discomfort* during the second/third wave. Çiçek Doğan et al. [36] reported limb pain, facial injuries and redness, sweating, weight loss, urticaria, and musculoskeletal problems. Luo et al. [40] described fogging of glasses, device-related injuries, hypoxia, palpitations, hypoglycemic symptoms, fatigue, and limitations associated with being unable to drink, eat, or leave while using PPE for prolonged periods. Chen et al. [35] included *physical distress* among frontline nurses’ experiences.

#### 3.3.3. Social and Family Dimension

The family and social sphere emerged mainly in pandemic-related studies. Fontanini et al. [37] described home isolation and fear of infecting family members; in the follow-up by Rossettini et al. [41], the subtheme *protecting my family* decreased significantly during the second/third wave compared with the first. Chen et al. [35] reported concerns about family as well as family and social support. Çiçek Doğan et al. [36] described fear of infecting family members, isolation, and social exclusion; Jiang et al. [26] included *family and home impact* among the work concerns. Luo et al. [40] reported difficulties returning home, concerns about family members, episodes of avoidance/exclusion upon returning to their communities, and support from family and other social levels.

#### 3.3.4. Professional Dimension

The narratives frequently situated distress within the professional role and concrete working conditions. In the blogs analyzed by Castro and Andrews [34] stress and burnout coexisted with frustrations related to teamwork, patients/families, and management. Forte and Pires [39] showed how fear, pressure, and fatigue were expressed alongside demands for PPE, professional recognition, decent working conditions, working hours, and salary. Rossettini et al. [41] documented the persistence of problematic working conditions across waves and an increase in the subtheme *despite everything, still devaluated*. Luo et al. [40] described the relationship between workload, PPE, conflicts with patients/families, support, recognition, and the meaning attributed to professional experience. Milicia et al. [38] identified constrained professional agency, compensation, inadequate nurse staffing, violence against nursing, and emotional burden. Overall, the corpus does not support a single empirical definition of distress but shows varying combinations of emotional, physical, social, and professional experiences.

### 3.4. Organizational Factors and Working Conditions Described as Sources of Distress

The primary studies included numerous references to organizational and working conditions. These elements are described here as problems within the work context reported by nurses or as conditions co-occurring within the same narratives; they are not interpreted as predictors or as causally demonstrated associations.

Castro and Andrews [34] identified *teamwork problems*, *challenging patients and families*, and *management issues* as sources of frustration; specific elements included understaffing, professional hierarchies, unprofessional behaviors, documentation workload, and unsafe work environments. Forte and Pires [39] documented demands concerning the availability of PPE and other work equipment, precarious conditions, working hours, salary, and professional rights. Fontanini et al. [37] described the rapid transformation of care environments, insufficient human and material resources, transfers to COVID-19 units, and protection-related problems; in the follow-up by Rossettini et al. [41], the subtheme *they are playing with our lives* increased during the second/third wave, whereas *working in a changing environment* decreased.

Çiçek Doğan et al. [36] identified shortages of staff and equipment, increased working hours and workload, financial difficulties, professional rights, administrative discrimination, and violence. Jiang et al. [26] documented workload, staffing, task completion and performance, working conditions and safety, pay and benefits, recognition and responsibilities, and future job situations. Luo et al. [40] reported shortages of staff and PPE, prolonged workload, infection risk, and difficulties in interactions with patients and family members. Milicia et al. [38], limited to the Reddit component, identified *Lack of Meaningful Recognition, Compensation, and Influence* and *Work Environment*, including constrained professional agency, compensation, inadequate nurse staffing, violence against nursing, and emotional burden.

The cross-study synthesis therefore shows recurring, although differently expressed and emphasized, issues related to staffing and workload, material resources and PPE, safety and organizational conditions, compensation and rights, recognition, professional agency, and violence/incivility.

### 3.5. Social Media as Spaces for Expression, Discussion, Support, and Professional Representation

Digital environments served not only as sources of data on work experiences but also as contexts in which nurses made narratives, demands, reflections, and representations of their professional role publicly visible. These functions were not explicitly investigated to the same extent in all studies.

Castro and Andrews [34] showed the use of anonymous/pseudonymous blogs to narrate working life longitudinally and share frustrations. Forte and Pires [39] described the use of Twitter and Instagram to encourage the public to stay at home, request PPE, draw attention to working conditions, and call for greater professional recognition. Fontanini et al. [37] made visible, through online narratives, both public recognition and perceptions of stigma and persistent devaluation; Rossettini et al. [41] showed how these narratives continued during subsequent waves and how social media were also used for messages directed at the public.

Chen et al. [35] provided some of the most explicit evidence of social media as a space for narration and support: online diaries were used to record experiences, interact with other users, and obtain self-support, with the themes *constructing a better self* and *constructing a strong support network*. Luo et al. [40] similarly described Weibo diaries as a form of expressive writing and self-regulation; the posts made visible national, social, team, and family support; attention from other users; and reflections on recognition and the public image of the profession. Çiçek Doğan et al. [36] documented communications directed at society concerning preventive behaviors, vaccination, and professional issues. AllNurses, in Jiang et al. [26], and Reddit, in Milicia et al. [38], functioned primarily as discussion environments in which concerns, work concerns, and well-being issues were shared. Evidence mapping of the main domains identified in the individual reports is presented in Table 3.

#### Differences Across Digital Environments

The included platforms differed in format, degree of anonymity, content length, and interaction structure, encompassing longitudinal blogs, public microblogs, Facebook pages and professional websites, forums, and comments. The corpus included Twitter and Instagram [39], Sina Weibo [40], and longitudinal data from Italian professional platforms [41]. However, no included article directly compared different platforms within a study design intended to test their effects. It is therefore not possible to conclude that one platform favors disclosure of distress, social support, or specific forms of professional representation more than another.

## 4. Discussion

This scoping review extends current knowledge on nurses’ occupational distress by examining how work-related experiences are represented in nurse-generated social media content. Rather than constituting an alternative measure of occupational distress, the digital narratives identified across the included studies offer a complementary perspective on how nurses articulate, contextualize, and publicly negotiate difficult working experiences outside researcher-defined settings. This distinction is important because occupational distress is increasingly conceptualized as a multidimensional phenomenon embedded in the interaction between individual experience and organizational conditions rather than as a single psychological outcome [5]. The contribution of the present review therefore lies less in identifying previously unrecognized manifestations of distress than in showing how established occupational concerns become visible within naturally occurring digital communication.

The multidimensional character of the experiences represented online is broadly consistent with the international nursing literature. Occupational stress has been associated with nurses’ psychological health [6], quality of working life and turnover intention [42], caring behaviours and health-related quality of life [43], while burnout has been conceptualized as a complex occupational phenomenon arising from interactions between individual and work-environment characteristics [44]. More recent evidence has similarly emphasized the interconnections among occupational stress, compassion fatigue, and work engagement [45], as well as the relationship between modifiable work stress factors and psychological health risk across nurses working in different countries [46]. Evidence concerning moral injury further demonstrates that ethically challenging work experiences may coexist with adverse mental health outcomes among nurses [47]. Viewed against this broader literature, the emotional, physical, social, and professional experiences represented in the included digital narratives appear to reflect interconnected dimensions of occupational strain rather than isolated phenomena.

At the same time, this convergence with conventional research should not obscure important conceptual differences. Occupational distress, burnout, stress, anxiety, moral distress, compassion fatigue, and related constructs cannot be treated as interchangeable merely because they co-occur in digital narratives. Recent conceptual work specifically characterizes occupational distress as a multidimensional work-related phenomenon while distinguishing it from related antecedents, manifestations, and consequences [5]. Likewise, burnout has its own theoretical and empirical literature and should not be inferred solely from colloquial use of the term in online communication [44]. Social media expressions such as “stress,” “burnout,” or “anxiety” therefore provide evidence about how occupational experiences are narrated and interpreted, but they cannot be assumed to correspond to diagnoses or scores obtained through validated psychometric instruments. This distinction is also consistent with evidence showing that subjective psychological burden may not necessarily parallel conventional clinical indicators [48]. This is particularly relevant when computational approaches classify sentiment or extract themes from large datasets: such outputs characterize the analyzed content rather than the prevalence, severity, or distribution of psychological states within the nursing workforce.

A central interpretive finding is the extent to which distress-related narratives are embedded in descriptions of the work environment. This is consistent with a substantial body of international evidence indicating that nurses’ occupational well-being is closely connected with organizational conditions. Integrative evidence has identified workload, staffing, time pressure, emotional demands, organizational constraints, and interpersonal difficulties as important demands within nursing work [12]. Occupational stress has also been associated with working conditions and organizational factors across different healthcare settings [7,19,20]. More broadly, organizational factors have been identified as important determinants of nurses’ workplace well-being [49], while large international studies have linked modifiable work stress factors with psychological health risk [46]. The recurrence of staffing, workload, resources, safety, recognition, professional agency, compensation, and organizational support within nurse-generated content therefore situates the present findings within an established international discussion about the structural determinants of nurses’ occupational well-being.

The Job Demands–Resources (JD-R) model provides a particularly useful framework for interpreting this convergence without imposing causal relationships on the evidence mapped in this review. The model proposes that occupational well-being reflects the dynamic balance between demands requiring sustained physical or psychological effort and resources that facilitate goal achievement, reduce demands, or support professional development [10,11]. Within nursing, integrative evidence has identified workload, time pressure, staffing constraints, emotional demands, role conflict, and adverse interpersonal experiences as relevant job demands, while autonomy, social support, leadership, recognition, and organizational resources may operate as job resources [12]. Research has further demonstrated relationships between job demands and resources and burnout and turnover intention among nurses [18], while more recent longitudinal evidence among healthcare professionals has examined whether job resources may buffer the harmful effects of demands on burnout complaints [16].

Importantly, the digital narratives mapped in this review should not be interpreted solely through a deficit-oriented perspective. Although organizational pressures were prominent, the included evidence also represented relational support, cooperation, self-support, professional commitment, meaning-making, and personal or professional growth. This is consistent with research showing that supportive clinical work environments are related to nurses’ motivation and well-being [17]. Leadership may be particularly relevant in this context. A recent systematic review examining leadership within the JD-R framework identified leadership as a potentially important resource in relation to nurses’ burnout and engagement [50], while recent evidence has also examined coordinator leadership in relation to burnout and nurses’ intention to leave [51]. Thus, the coexistence of demands and resources within online narratives is theoretically meaningful: social media may capture not only what nurses experience as burdensome but also the interpersonal and professional resources through which those experiences are interpreted and managed.

Workplace safety represents another area in which the findings intersect with international evidence. Workplace violence has been documented as an important occupational issue in nursing and has been examined in relation to work characteristics and professional experience [15]. Particularly severe forms of workplace violence may have substantial psychological and professional consequences; for example, a systematic review has documented sexual violence against nurses working in inpatient psychiatric settings and its associated outcomes and risk factors [52]. Similarly, workload has been examined in relation to moral distress, burnout, and turnover in intensive care settings [53]. Other work processes may also contribute to nurses’ perceived burden: time-motion and stress-monitoring research has demonstrated stress associated with nursing activities and electronic health record use [54]. These studies provide an important external context for interpreting the organizational concerns represented online, while remaining methodologically distinct from the evidence included in this scoping review.

The predominance of pandemic-related evidence requires particular consideration. COVID-19 created an exceptional combination of infection risk, resource scarcity, rapidly changing organizational arrangements, increased workload, social isolation, and heightened public attention toward nursing. Previous research specifically examining nurses’ social media use during the pandemic showed that digital platforms became important environments for communicating professional experiences and pandemic-related concerns [21,22]. Accordingly, the prominence of PPE, infection risk, isolation, rapidly changing work environments, and public recognition within the present corpus should not automatically be generalized to routine nursing practice. At the same time, the presence of occupational concerns in pre-pandemic nursing narratives suggests that social media representations of professional strain cannot be reduced entirely to the COVID-19 emergency. Rather, the pandemic appears to have intensified and made particularly visible organizational problems that overlap with longstanding concerns in the nursing workforce.

The geographical heterogeneity of the included studies should also be considered when interpreting these findings. Differences in healthcare contexts, working conditions, and social media use may shape how occupational experiences are expressed online. However, as the included studies were not designed for cross-country comparisons, any geographical variation should be interpreted cautiously and cannot be separated from methodological and platform-related differences.

This temporal dimension is especially relevant because occupational narratives are unlikely to remain stable across changing organizational and societal contexts. The longitudinal evidence included in the review indicates that the salience of specific experiences can change across different phases of a crisis. This reinforces the value of treating social media data as temporally situated rather than as static representations of professional experience. It also suggests a potentially useful role for longitudinal digital research in examining how professional concerns evolve alongside changes in workload, staffing, policy, public discourse, and organizational conditions. However, such approaches require independent datasets and careful attention to changes in platform use and user populations over time.

Beyond their substantive content, social media narratives have methodological value because they are often generated outside conventional research encounters. Traditional questionnaires and interviews remain essential for assessing occupational health outcomes, but they inevitably structure participation through researcher-defined questions, constructs, and settings. Social media can provide access to naturally occurring narratives in which users decide what experiences are worth communicating and how these experiences should be framed. Methodological literature has recognized this potential while emphasizing that social media research presents specific challenges related to sampling, interpretation, data access, and validity [24,55]. Consequently, digital narratives should be understood as complementary rather than competing sources of evidence.

This complementarity is particularly important because what becomes visible online is shaped by processes of self-selection and self-presentation. Social media users actively construct representations of themselves for perceived audiences, and these processes may affect which occupational experiences are disclosed and how they are narrated [56]. Nurses who publicly discuss distress may systematically differ from those who remain silent, while anonymity, professional identity, audience expectations, community norms, and platform affordances may influence communication. Large datasets do not resolve these problems: increasing the number of posts may improve the description of a digital corpus but does not transform that corpus into a representative sample of nurses. This distinction is fundamental when considering the potential use of social media for occupational health or workforce research.

The social and professional functions of digital environments also deserve attention. Social media use among healthcare professionals has long been associated with the development of virtual communities and opportunities for professional interaction and knowledge exchange [57]. More recent research suggests that social media can provide insight into careers and professional experiences within a changing world of work [58], while its use in professional organizations may have both supportive and demanding implications for the workforce [59]. Within nursing specifically, digital technologies may interact with professional identity, autonomy, and relations of power [60]. The narration, advocacy, support, public communication, and professional self-representation identified in the present review can therefore be situated within a broader literature that understands digital environments as active contexts for professional identity and interaction rather than merely repositories of research data.

This perspective may also help explain apparently contradictory representations of nursing within digital narratives. Professional pride, commitment, solidarity, and meaning-making can coexist with exhaustion, frustration, perceived devaluation, and organizational dissatisfaction. Such coexistence should not necessarily be interpreted as inconsistency. Rather, it may reflect the complexity of professional identity in contexts where nurses simultaneously derive meaning from their work and experience organizational conditions that threaten their well-being. Digital environments may make this tension particularly visible because they permit professional experience to be narrated simultaneously to peers, the public, and broader professional communities.

### 4.1. Ethical Aspects of Social Media Data Use

Ethical and data-governance practices varied across the primary studies. Forte and Pires [39] considered freely accessible social media content as data not requiring ethical approval, while stating that author anonymity was ensured. Fontanini et al. [37] and Rossettini et al. [41] reported anonymization of narratives and removal of identifying information relating to nurses and healthcare facilities while using publicly available content without individual consent. Luo et al. [40], by contrast, obtained approval from an ethics committee, followed guidelines for online research, anonymized individuals, hospitals, and sensitive locations, deleted user information after analysis, and restructured translated quotations to reduce traceability.

This variability confirms that the mere public availability of content does not resolve issues of contextual privacy, consent, traceability, and governance. The possibility of tracing a textual quotation back to an account, particularly when the content concerns emotional distress or sensitive working conditions, requires specific caution. In the present scoping review, the unit of analysis consisted of published articles: no new posts were collected, no users were contacted, and no attempts were made to identify the original authors.

### 4.2. Implications for Practice and Policy

The implications should remain proportionate to the nature of the evidence. The nine articles allow the identification of recurring problems, signals, and modes of representation but do not allow conclusions to be drawn regarding the effectiveness of organizational interventions or the strength or causality of relationships between working conditions and distress. This limitation is particularly relevant because no formal critical appraisal was conducted and because the included studies are predominantly descriptive, qualitative, or computational-exploratory.

At the level directly supported by the corpus, nurse-generated content can make perceived problems related to staffing, workload, PPE and resources, safety, compensation, recognition, professional agency, violence/incivility, and support visible. It can also show how nurses publicly process their experiences through narration, advocacy, requests for help, expressive writing, and professional representation. These observations are hypothesis-generating and do not, by themselves, provide a basis for prescribing interventions.

The rationale for interventions addressing staffing, workload, leadership, autonomy, support, and organizational conditions derives from the broader nursing literature and from studies specifically designed to evaluate their effects, not from the nine articles mapped in this scoping review alone. The present synthesis can therefore primarily contribute to identifying problems and dimensions deserving further investigation and to suggesting which qualitative signals might be integrated with independent data sources.

The use of social media content as a complementary source for identifying emerging themes is conceptually distinct from workforce surveillance. Posts do not constitute a representative sample of the nursing population and do not provide a basis for inferring the prevalence, severity, or distribution of distress. Potential organizational or policy applications raise issues concerning privacy, representativeness, access, governance, reporting, identification, surveillance, and individual assessment of nurses, consistent with the methodological cautions already highlighted by Takats et al. [55].

### 4.3. Limitations and Directions for Future Research

The findings should be interpreted in light of several limitations.

First, the concept was deliberately operationalized broadly to capture a terminologically heterogeneous literature. Expressions of distress, adjacent constructs, working conditions, and potential outcomes were kept conceptually distinct, but their inclusion within the same mapping inevitably introduces heterogeneity and requires caution when synthesizing non-equivalent phenomena.

Second, the corpus remains limited to nine articles and contains substantial overlap between Fontanini et al. [37] and Rossettini et al. [41] for the 380 first-wave narratives. The publications also differ in platform, data format, period, dataset size, and analytical approach. The identified domains should therefore be regarded as patterns emerging from this corpus rather than as a definitive taxonomy of occupational distress on social media.

Third, the corpus is heavily weighted toward the COVID-19 pandemic: seven articles are specifically COVID-19-related, and one additional study, Milicia et al. [38], covers a period spanning the pandemic. Infection risk, PPE, service reorganization, and isolation may have shaped experiences and digital communication in exceptional ways. Transferability to ordinary and post-pandemic contexts is therefore limited.

Fourth, geographical coverage remains limited and unevenly documented. Professional culture, service organization, employment regulation, and social media practices may vary substantially across healthcare systems and online communities.

Fifth, no language filters were applied in the search strategies, but eligibility was restricted to articles published in English or Italian. Grey literature was not included, and the electronic search was conducted in PubMed, CINAHL Complete, and Scopus without PsycINFO or Web of Science. The fact that backward and forward citation searching identified three additional eligible reports not retrieved through the database searches indicates that the electronic search alone did not identify all reports subsequently included in the review.

Sixth, the requirement that content be nurse-generated and, in multiprofessional studies, separately identifiable and analyzable may have excluded relevant research using mixed datasets. Moreover, the way in which professional identity was determined was not uniform: some studies used certified accounts or profile screening, others attributed content to nurses according to procedures described in the primary study, whereas Forte and Pires [39] defined the publications as originating from nursing professionals without further detailing the individual verification procedure.

Seventh, no formal assessment of methodological quality or risk of bias was performed. Consequently, the synthesis does not allow conclusions regarding certainty of evidence, prevalence of distress, causality, or intervention effectiveness. Differences in methods—from qualitative analysis to topic modelling and sentiment analysis—also introduce different assumptions and interpretive limitations.

Finally, social media data are subject to self-selection and platform-specific influences. Nurses who choose to post may differ from those who do not; anonymity, perceived audience, community norms, format, and self-presentation may influence what is shared. Even very large datasets do not automatically overcome this representativeness limitation.

Future research should expand the evidence in post-pandemic and more geographically diverse contexts, describe procedures for identifying authors as nurses more transparently, and adopt explicit ethical standards for contextual privacy and traceability. Comparative studies designed to assess the influence of platform characteristics are also needed, as are mixed-methods approaches integrating social media content with interviews, validated instruments, organizational indicators, or workforce data. Longitudinal studies, following the model of Rossettini et al. [41] but using independent datasets and longer periods, could clarify how narratives change in relation to organizational and historical contexts.

## 5. Conclusions

The present scoping review identified nine articles showing how nurse-generated social media content makes different representations of occupational distress and work-related experiences observable across psychological and emotional, physical, social and family, and professional-organizational dimensions. Across the included evidence, distress was represented alongside recurrent working and organizational conditions, including staffing and workload, availability of PPE and material resources, safety, compensation and professional rights, recognition, professional agency, and violence or incivility. These co-occurrences are descriptive and do not establish prevalence, statistical associations, or causal relationships.

The findings also show that social media can function as spaces in which nurses narrate and discuss work-related experiences, engage in expressive writing and supportive interactions, communicate with wider publics, and represent their professional role. The included evidence also illustrates how these narratives may vary over time and across changing work and crisis contexts. Social media content therefore provides access to situated digital narratives of occupational experiences rather than a representative account of the nursing workforce.

The available evidence remains limited and methodologically heterogeneous, with most studies conducted in the context of the COVID-19 pandemic and two publications drawing on partially overlapping datasets. The interpretation of these digital narratives is also shaped by self-selection, limited representativeness, and issues related to contextual privacy, identifiability, and data governance. Within these boundaries, the reviewed literature maps how occupational distress and related working conditions are represented in nurse-generated social media content, while the extent to which these patterns extend across post-pandemic periods, geographical contexts, platforms, and independent datasets remains uncertain.

## Figures and Tables

**Figure 1:**
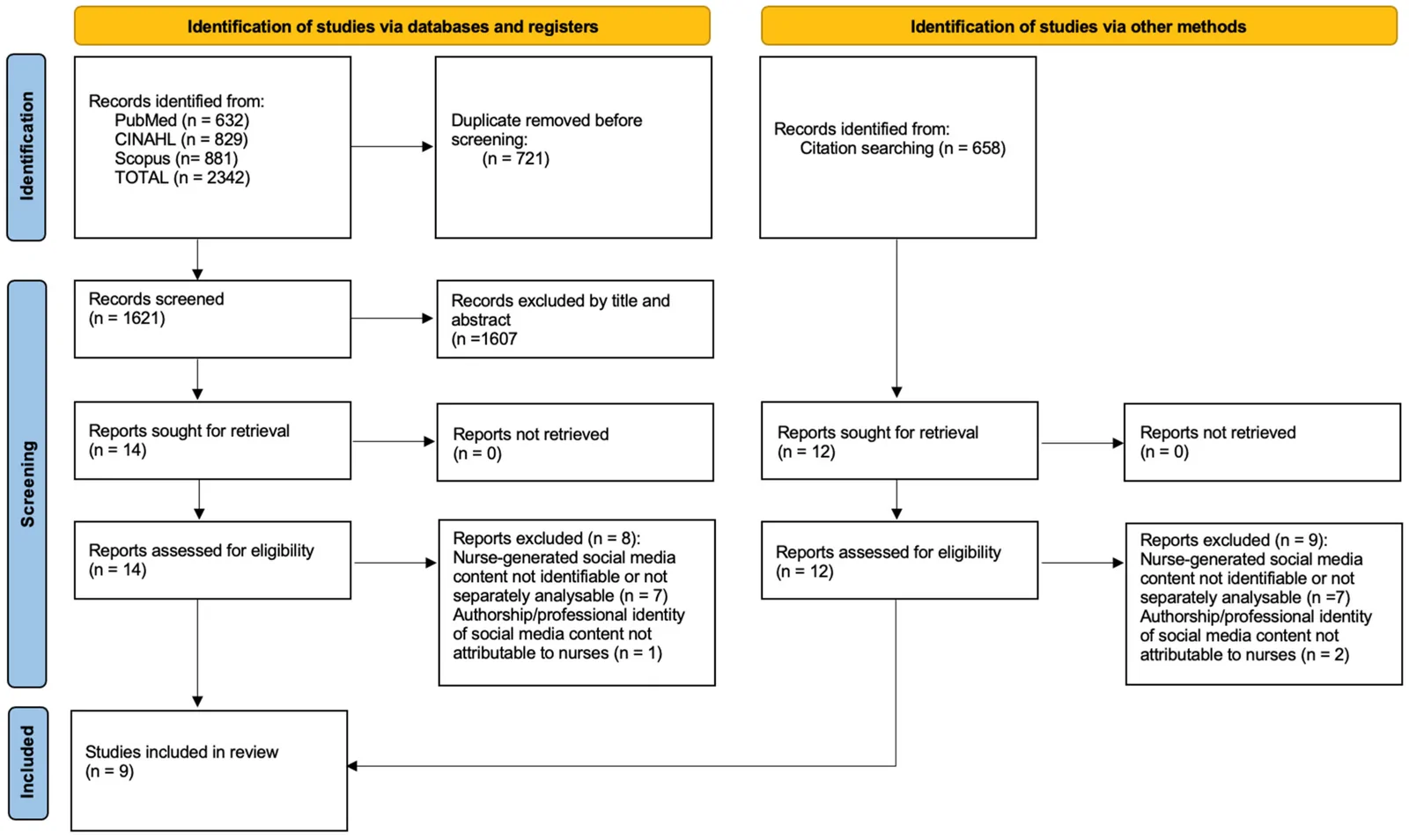
PRISMA flow diagram.

**Table 1:** Eligibility criteria according to the Population–Concept–Context (PCC) framework.

PCC	Operational Definition	Inclusion Criteria	Main Exclusions
**Population**	Nurses	Content attributed to nurses according to the procedures reported in the primary studies, irrespective of clinical specialty, healthcare setting, professional experience, or geographical location. In multiprofessional studies, nurse-specific data had to be identifiable and analyzable separately.	Content generated by patients, caregivers, nursing students, organizations, media outlets, or other professional groups when nurse-specific data were not separately identifiable.
**Concept**	Occupational distress and work-related experiences characterized by psychological, emotional, professional, or organizational strain	Expressions of distress and adjacent work-related constructs; working and organizational conditions described as sources or contributing factors; and related constructs or potential occupational outcomes, when relevant to the Concept of the review.	Phenomena unrelated to nurses’ work experiences or inconsistent with the Concept of the review.
**Context**	Social media content generated directly by nurses	Textual, audiovisual, or multimodal content shared through social media and online environments for interaction/discussion.	Use of social media exclusively for recruitment, questionnaire administration, intervention delivery, or information dissemination, without analysis of nurse-generated content.

**Table 2:** Characteristics of the included publications/reports.

Publication/Report	Title	Geographical Context	Period	Platform/ Source	Sample/ Data Type	Identification of Nursing Content	Analytical Approach	COVID-19	Main Domains
Castro & Andrews [34]	Nursing lives in the blogosphere: A thematic analysis of anonymous online nursing narratives	United States. All four bloggers were American nurses; specific institutions were not identified because the blogs were anonymous.	2014	Publicly accessible nursing blogs	520 entries from 4 blogs	Anonymous/pseudonymous authors identified by the primary study as nurses from four different specialties; blogs selected from lists recommended by nursing organizations	Qualitative description; inductive thematic coding	No	Stress/burnout; teamwork; patients/families; management
Forte & Pires [39]	Nursing appeals on social media in times of coronavirus	Brazilian context, but geographical provenance of individual posts is not explicitly established in the reported sampling procedure. The article concerns nursing appeals analysed in Portuguese-language Twitter and Instagram content; the authors are based in Brazil.	11–20 March 2020	Twitter and Instagram	295 publications: 101 Twitter; 194 Instagram	Publications defined by the authors as produced by nursing professionals; retrieved using #nursing AND #coronavirus; professional verification procedure not further detailed	Qualitative documentary study; thematic content analysis using ATLAS.ti	Yes	PPE; working conditions; pressure/distress; workday; salary/rights; professional recognition
Fontanini et al. [37] *	Italian Nurses’ experiences during the COVID-19 pandemic: a qualitative analysis of internet posts	Italy. Inclusion was restricted to nurses working in Italy. Among the 200 narratives for which geographical information was available, 67 (33.5%) were from nurses working in Lombardy; geographical information was therefore not available for all 380 narratives.	23 February–3 May 2020	Facebook and professional nursing websites/pages	380 first-wave narratives; 2510 quotes	Narratives posted by nurses working in Italy; nursing students, other healthcare professionals, and nurses working abroad excluded	Qualitative descriptive study; content analysis	Yes	Emotional/physical distress; working conditions; isolation; recognition/stigma; growth
Rossettini et al. [41] *	Italian nurses’ experiences of the COVID-19 pandemic through social media: A longitudinal mixed methods study of Internet posts	Italy. Narratives were required to be produced by nurses working in Italy; nurses working abroad were excluded. The first-wave component is the same Italian corpus analysed by Fontanini et al. [37]	23 February–3 May 2020; 13 October 2020–23 May 2021	Facebook and five professional nursing websites/pages	380 first-wave narratives already analyzed in Fontanini et al. (2021) [37] + 161 new second/third-wave narratives	Narratives produced by nurses working in Italy; students, other professionals, and content not referring to direct experience excluded	Longitudinal mixed-methods; extension of first-wave corpus with quantitative/qualitative comparison across waves	Yes	Emotional/physical distress; working conditions; family; recognition/devaluation; aggression; professional future; vaccines
Chen et al. [35]	The experiences of frontline nurses in Wuhan: A qualitative analysis of nurse online diaries during the COVID-19 pandemic	Wuhan, Hubei, China. Nurses were working in Wuhan pandemic hospitals or Fangcang shelter hospitals. The 12 nurses originated from hospitals in different provinces of China, but the individual provinces are not specified in the article.	23 January–8 April 2020	Sina Microblog	205 diary entries; 12 nurses	Certified frontline nurses working in Wuhan pandemic hospitals or Fangcang shelter hospitals; professional identities certified on Sina Microblog	Qualitative content analysis	Yes	Physical/psychological distress; self-support; social support; growth; professionalism
Çiçek Doğan et al. [36]	A Qualitative Analysis Using Social Listening Method: Turkish Nurses’ Experiences in Coronavirus Disease 2019 Pandemic	Turkey. The study explicitly concerns Turkish nurses and Turkish-language Twitter content. A more specific geographical distribution of the nurse users is not reported in the full text.	11 March 2020–11 March 2022	Twitter	9330 tweets; 1051 meaning units	Candidate users identified using Turkish expressions indicating “I am a nurse”; accounts/tweets manually reviewed; news sources, student nurses, retired nurses, inactive users, fake accounts, and nurses reporting that they were not employed were excluded; 3823 nurse users identified	Qualitative content analysis/social listening	Yes	Stress/fear; physical health; staffing/equipment; rights/finances; violence/discrimination; social life
Jiang et al. [26]	Nurses’ Work Concerns and Disenchantment During the COVID-19 Pandemic: Machine Learning Analysis of Web-Based Discussions	Predominantly United States context, but individual contributors’ geographical locations were not established. The authors describe AllNurses discussions as involving primarily US-based colleagues; the dataset itself does not provide a verified geographical location for every poster.	March 2020–April 2021	AllNurses, “COVID, Disaster, Pandemic” forum	14,060 posts	Content collected from a professional nursing forum; analysis focused on nurse-generated discussions	LDA topic modelling + qualitative human-in-the-loop analysis	Yes	Workload; staffing; safety; pay/benefits; recognition/responsibilities; burnout; family
Luo et al. [40]	Understanding early experiences of Chinese frontline nurses during the COVID-19 pandemic: A text mining and thematic analysis of social media information	China. Authentication information for the 97 nurses covered 14 provincial-level locations: Hubei 73 (75.26%), Liaoning 5 (5.15%), Anhui 3 (3.09%), Beijing 3 (3.09%), Guangdong 2 (2.06%), Guizhou 2 (2.06%), Zhejiang 2 (2.06%), and one each (1.03%) from Hunan, Jilin, Jiangxi, Ningxia, Shandong, Shanghai, and Yunnan. The narratives included nurses supporting frontline work in Wuhan as well as nurses working in other Chinese settings.	January–April 2020	Sina Weibo	5112 posts from 97 nurses; qualitative subset of 26 nurses	Profile search using “nurse”; official certification or self-identification supplemented by manual verification	Explanatory sequential mixed-methods; text mining/sentiment analysis + thematic analysis	Yes	Psychological/physical distress; staffing/PPE; support; self-regulation/expressive writing; recognition/meaning-making
Milicia et al. [38]	Are They Aligned? A Qualitative Analysis of Social Media–Based Nurse Well-Being Concerns and Well-Being Programs	Not ascertainable for the Reddit sample from the full text. The geographical location of individual Reddit contributors was not reported/verified; therefore, the Reddit dataset should not be labelled as US-based solely from author affiliations or the locations of the healthcare systems interviewed in the other component of the study.	2018–2022	Reddit	Source corpus of 78,122 comments; 120 comments selected for qualitative analysis	Nurse-specific Reddit forum; selected comments were from users self-reporting as registered nurses; professional identity could not be independently verified	Mixed-methods primary study; only findings from the social-media component considered for this review	Period spans COVID-19; not exclusively COVID-19	Recognition; compensation; constrained professional agency; staffing; violence; emotional burden; misinformation

Note. * Fontanini et al. [37] and Rossettini et al. [41] are two publications with a partially overlapping corpus. Fontanini et al. [37] analyzed 380 narratives from the first pandemic wave. Rossettini et al. [41] incorporated the same 380 first-wave narratives and added 161 new narratives from the second/third wave, allowing a longitudinal comparison. Both publications were included because they provide complementary analytical contributions; however, findings derived from the 380 shared narratives were not interpreted as independent evidence in the synthesis.

**Table 3:** Evidence mapping of findings across the included reports.

Synthesis Area/Mapped Thematic Area		Castro & Andrews [34]	Forte & Pires [39]	Fontanini et al. [37] *	Rossettini et al. [41] *	Chen et al. [35]	Çiçek Doğan et al. [36]	Jiang et al. [26]	Luo et al. [40]	Milicia et al. [38]
**Expressions and experiences related to distress**	Stress, burnout and/or emotional distress	✓	✓	✓	✓	✓	✓	✓	✓	✓
	Fear and/or risk of infection	**✗**	✓	✓	✓	✓	✓	✓	✓	**✗**
	Physical manifestations	**✗**	✓	✓	✓	✓	✓	**✗**	✓	**✗**
	Family impact and/or isolation	**✗**	**✗**	✓	✓	✓	✓	✓	✓	**✗**
**Reported working and organizational conditions**	Staffing and/or workload	✓	✓	✓	✓	**✗**	✓	✓	✓	✓
	PPE and/or material resources	**✗**	✓	✓	✓	**✗**	✓	✓	✓	**✗**
	Management, policies and/or organizational conditions	✓	✓	✓	✓	**✗**	✓	✓	**✗**	✓
	Compensation/pay and/or rights	**✗**	✓	**✗**	✓	**✗**	✓	✓	**✗**	✓
	Professional agency, autonomy and/or responsibility	**✗**	**✗**	**✗**	**✗**	**✗**	**✗**	✓	**✗**	✓
	Violence, incivility and/or aggression	✓	**✗**	✓	✓	**✗**	✓	**✗**	**✗**	✓
	Professional recognition and/or devaluation	**✗**	✓	✓	✓	**✗**	**✗**	✓	✓	✓
	Stigma, exclusion and/or misinformation	**✗**	**✗**	✓	✓	**✗**	✓	✓	✓	✓
**Other content and functions represented in digital narratives**	Pride, commitment and/or professionalism	✓	**✗**	✓	✓	✓	**✗**	**✗**	✓	**✗**
	Social support and/or supportive online interactions	✓	**✗**	**✗**	✓	✓	**✗**	**✗**	✓	**✗**
	Narration as self-support, debriefing and/or expressive writing	**✗**	**✗**	**✗**	**✗**	✓	**✗**	**✗**	✓	**✗**
	Personal/professional growth and/or meaning-making	**✗**	**✗**	✓	✓	✓	**✗**	**✗**	✓	**✗**

✓ = domain explicitly represented in the findings of the publication; **✗** = domain not identified with sufficient explicitness for mapping. For categories aggregated using “and/or,” the symbol ✓ indicates that at least one component of the domain was explicitly represented in the findings of the publication; inclusion within the same category does not imply conceptual equivalence among the different constructs. Columns represent publications/reports and not necessarily independent datasets. Note * Fontanini et al. [37] and Rossettini et al. [41] share the 380 narratives from the first wave; Rossettini et al. [41] add 161 narratives from the second/third wave and a longitudinal comparison. Therefore, the presence of the same domain in both columns should not be interpreted as evidence derived from two independent datasets. The matrix is intended exclusively for descriptive evidence mapping and was not used to calculate frequencies, prevalence, importance, strength of evidence, or the number of independent studies supporting individual domains.

## Data Availability

No new data were created or analyzed in this study. Data sharing is not applicable to this article.

## Supplementary Materials

The following supporting information can be downloaded at https://www.mdpi.com/article/10.3390/healthcare14193146/s1, Table S1: Full search strategies; PRISMA-ScR Checklist.
