# Supplementary material for: Occupational Distress Among Nurses Through Social Media Narratives: A Scoping Review
Source: Healthcare (Basel). 2026 Sep 22;14(19):3146. doi: 10.3390/healthcare14193146 (PMC13665435; doi:10.3390/healthcare14193146)
Supplement: Supplementary file 1 [file healthcare-14-03146-s001.zip › Table S1. Full search strategies_08.09.pdf]

Supplemental material. Search strategy

<table>
<tr><td colspan="3">Search strategy PubMed. Date: 02.09.2026., Filters: none

Total number of hits: 632

(((("Nurses"[Mesh]) OR ("Nursing Staff"[Mesh])) OR (Nurse* OR "nursing staff")) AND (("Social Media"[Mesh]) OR ("social media" OR "social networking" OR "social network*" OR "online communit*" OR "online forum*" OR "web-based forum*" OR "discussion forum*" OR TikTok OR Instagram OR Facebook OR Twitter OR Reddit OR YouTube OR blog* OR vlog*))) AND ((((("Occupational Stress"[Mesh]) OR ("Burnout, Professional"[Mesh])) OR ("Burnout, Psychological"[Mesh])) OR ("Compassion Fatigue"[Mesh])) OR ("Emotional Exhaustion"[Mesh])) OR (Burnout OR "occupational distress" OR "occupational stress" OR "work-related stress" OR "work related stress" OR "job stress" OR "workplace stress*" OR "work concern*" OR "moral distress" OR "moral injury" OR "emotional exhaustion" OR "compassion fatigue" OR "psychological distress" OR "psychosocial health" OR frustration OR "well-being" OR wellbeing OR "workplace violence" OR discrimination OR understaff* OR "staffing shortage*" OR workload OR "work load" OR "professional autonomy" OR recognition OR "job dissatisfaction" OR "intention to leave"))</td></tr>
<tr><td>Block 1 - Nurses</td><td>Block 2 - Social media and online discussion environments</td><td>Block 3 - Occupational distress and related work-related phenomena</td></tr>
<tr><td>"Nurses"[Mesh]
OR "Nursing Staff"[Mesh]

Nurse*
OR "nursing staff"</td><td>"Social Media"[Mesh]

"social media"
"social networking"
"social network*"
"online communit*"
"online forum*"
"web-based forum*"
"discussion forum*"
TikTok
Instagram
Facebook
Twitter
Reddit
YouTube
blog*
vlog*</td><td>"Occupational Stress"[Mesh]
"Burnout, Professional"[Mesh]
"Burnout, Psychological"[Mesh]
"Compassion Fatigue"[Mesh]
"Emotional Exhaustion"[Mesh]

burnout
"occupational distress"
"occupational stress"
"work-related stress"
"work related stress"
"job stress"
"workplace stress*"
"work concern*"
"moral distress"
"moral injury"
"emotional exhaustion"
"compassion fatigue"
"psychological distress"
"psychosocial health"
frustration
"well-being"
wellbeing
"workplace violence"
discrimination
understaff*
"staffing shortage*"
workload</td></tr>
</table>

Supplemental material. Search strategy

		**"work load"**<br>**"professional autonomy"**<br>**recognition**<br>**"job dissatisfaction"**<br>**"intention to leave"**

**PUBMED**		
**ID#**	**Search Term**	**Results**
1	"Nurses"[Mesh]	**106,809**
2	"Nursing Staff"[Mesh]	**74,324**
3	Nurse* OR "nursing staff"	**567,063**
4	#1 OR #2 OR #3	**567,068**
5	"Social Media"[Mesh]	**22,869**
6	"social media" OR "social networking" OR "social network*" OR "online communit*" OR "online forum*" OR "web-based forum*" OR "discussion forum*" OR TikTok OR Instagram OR Facebook OR Twitter OR Reddit OR YouTube OR blog* OR vlog*	**105,200**
7	#5 OR #6	**105,200**
8	"Occupational Stress"[Mesh]	**26,890**
9	"Burnout, Professional"[Mesh]	**21,794**
10	"Burnout, Psychological"[Mesh]	**22,802**
11	"Compassion Fatigue"[Mesh]	**1,495**
12	"Emotional Exhaustion"[Mesh]	**342**
13	Burnout OR "occupational distress" OR "occupational stress" OR "work-related stress" OR "work related stress" OR "job stress" OR "workplace stress*" OR "work concern*" OR "moral distress" OR "moral injury" OR "emotional exhaustion" OR "compassion fatigue" OR "psychological distress" OR "psychosocial health" OR frustration OR "well-being" OR wellbeing OR "workplace violence" OR discrimination OR understaff* OR "staffing shortage*" OR workload OR "work load" OR "professional autonomy" OR recognition OR "job dissatisfaction" OR "intention to leave")	**1,359,755**
14	#8 OR #9 OR #10 OR #11 OR #12 OR #13	**1,359,755**
15	#4 AND #7 AND #14	**632**

Supplemental material. Search strategy

Search strategy CINAHL Complete. Date: 02.09.2026., Filters: none

Total number of hits: 829

(MH "Nurses+" OR nurse* OR "nursing staff") AND (MH "Social Media+" OR "social media" OR "social networking" OR "social network*" OR "online communit*" OR "online forum*" OR "web-based forum*" OR "discussion forum*" OR TikTok OR Instagram OR Facebook OR Twitter OR Reddit OR YouTube OR blog* OR vlog*) AND (MH "Stress, Occupational+" OR Burnout OR "occupational distress" OR "occupational stress" OR "work-related stress" OR "work related stress" OR "job stress" OR "workplace stress*" OR "work concern*" OR "moral distress" OR "moral injury" OR "emotional exhaustion" OR "compassion fatigue" OR "psychological distress" OR "psychosocial health" OR frustration OR "well-being" OR wellbeing OR "workplace violence" OR discrimination OR understaff* OR "staffing shortage*" OR workload OR "work load" OR "professional autonomy" OR recognition OR "job dissatisfaction" OR "intention to leave")

Block 1 - Nurses	Block 2 - Social media and online discussion environments	Block 3 - Occupational distress and related work-related phenomena
MH "Nurses+"<br><br>**Nurse***<br>**"nursing staff"**	MH "Social Media+"<br><br>**"social media"**<br>**"social networking"**<br>**"social network*"**<br>**"online communit*"**<br>**"online forum*"**<br>**"web-based forum*"**<br>**"discussion forum*"**<br>**TikTok**<br>**Instagram**<br>**Facebook**<br>**Twitter**<br>**Reddit**<br>**YouTube**<br>**blog***<br>**vlog***	MH "Stress, Occupational+"<br><br>**burnout**<br>**"occupational distress"**<br>**"occupational stress"**<br>**"work-related stress"**<br>**"work related stress"**<br>**"job stress"**<br>**"workplace stress*"**<br>**"work concern*"**<br>**"moral distress"**<br>**"moral injury"**<br>**"emotional exhaustion"**<br>**"compassion fatigue"**<br>**"psychological distress"**<br>**"psychosocial health"**<br>**frustration**<br>**"well-being"**<br>**wellbeing**<br>**"workplace violence"**<br>**discrimination**<br>**understaff***<br>**"staffing shortage*"**<br>**workload**<br>**"work load"**<br>**"professional autonomy"**<br>**Recognition**<br>**"job dissatisfaction"**<br>**"intention to leave"**

Supplemental material. Search strategy

CINAHL Complete		
**ID#**	**Search Term**	**Results**
1	MH "Nurses+"	**268,285**
2	nurse* OR "nursing staff"	**646,793**
3	S1 OR S2	**652,914**
4	MH "Social Media+"	**31,429**
5	"social media" OR "social networking" OR "social network*" OR "online communit*" OR "online forum*" OR "web-based forum*" OR "discussion forum*" OR TikTok OR Instagram OR Facebook OR Twitter OR Reddit OR YouTube OR blog* OR vlog*	**75,602**
6	S4 OR S5	**75,602**
7	MH "Stress, Occupational+"	**43,793**
8	Burnout OR "occupational distress" OR "occupational stress" OR "work-related stress" OR "work related stress" OR "job stress" OR "workplace stress*" OR "work concern*" OR "moral distress" OR "moral injury" OR "emotional exhaustion" OR "compassion fatigue" OR "psychological distress" OR "psychosocial health" OR frustration OR "well-being" OR wellbeing OR "workplace violence" OR discrimination OR understaff* OR "staffing shortage*" OR workload OR "work load" OR "professional autonomy" OR recognition OR "job dissatisfaction" OR "intention to leave"	**373,949**
9	S7 OR S8	**379,450**
10	S3 AND S6 AND S9	**829**

Search strategy SCOPUS. Date: 02.09.2026., Filters: none

Total number of hits: 881

(TITLE-ABS-KEY (nurse* OR "nursing staff") AND TITLE-ABS-KEY (("social media" OR "social networking" OR "social network*" OR "online communit*" OR "online forum*" OR "web-based forum*" OR "discussion forum*" OR tiktok OR instagram OR facebook OR twitter OR reddit OR youtube OR blog* OR vlog*)) AND TITLE-ABS-KEY ((burnout OR "occupational distress" OR "occupational stress" OR "work-related stress" OR "work related stress" OR "job stress" OR "workplace stress*" OR "work concern*" OR "moral distress" OR "moral injury" OR "emotional exhaustion" OR "compassion fatigue" OR "psychological distress" OR "psychosocial health" OR frustration OR "well-being" OR wellbeing OR "workplace violence" OR discrimination OR understaff* OR "staffing shortage*" OR workload OR "work load" OR "professional autonomy" OR recognition OR "job dissatisfaction" OR "intention to leave")))

Supplemental material. Search strategy

Block 1 - Nurses	Block 2 - Social media and online discussion environments	Block 3 - Occupational distress and related work-related phenomena
**Nurse***<br>**"nursing staff"**	**"social media"**<br>**"social networking"**<br>**"social network*"**<br>**"online communit*"**<br>**"online forum*"**<br>**"web-based forum*"**<br>**"discussion forum*"**<br>**TikTok**<br>**Instagram**<br>**Facebook**<br>**Twitter**<br>**Reddit**<br>**YouTube**<br>**blog***<br>**vlog***	**burnout**<br>**"occupational distress"**<br>**"occupational stress"**<br>**"work-related stress"**<br>**"work related stress"**<br>**"job stress"**<br>**"workplace stress*"**<br>**"work concern*"**<br>**"moral distress"**<br>**"moral injury"**<br>**"emotional exhaustion"**<br>**"compassion fatigue"**<br>**"psychological distress"**<br>**"psychosocial health"**<br>**frustration**<br>**"well-being"**<br>**wellbeing**<br>**"workplace violence"**<br>**discrimination**<br>**understaff***<br>**"staffing shortage*"**<br>**workload**<br>**"work load"**<br>**"professional autonomy"**<br>**Recognition**<br>**"job dissatisfaction"**<br>**"intention to leave"**

**SCOPUS**		
**ID#**	**Search Term [Article title, Abstract, Keywords]**	**Results**
1	nurse* OR "nursing staff"	661,565
2	"social media" OR "social networking" OR "social network*" OR "online communit*" OR "online forum*" OR "web-based forum*" OR "discussion forum*" OR TikTok OR Instagram OR Facebook OR Twitter OR Reddit OR YouTube OR blog* OR vlog*	571,965
3	Burnout OR "occupational distress" OR "occupational stress" OR "work-related stress" OR "work related stress" OR "job stress" OR "workplace stress*" OR "work concern*" OR "moral distress" OR "moral injury" OR "emotional exhaustion" OR "compassion fatigue" OR "psychological distress" OR "psychosocial health" OR frustration OR "well-being" OR	3,054,294

	wellbeing OR "workplace violence" OR discrimination OR understaff* OR "staffing shortage*" OR workload OR "work load" OR "professional autonomy" OR recognition OR "job dissatisfaction" OR "intention to leave"	
4	1 AND 2 AND 3	881
